# Proteomic Variation in the Oral Secretion of *Spodoptera exigua* and *Spodoptera littoralis* Larvae in Response to Different food Sources

**DOI:** 10.1007/s10886-025-01571-9

**Published:** 2025-01-24

**Authors:** Elena García-Marín, Jordi Gamir, Cristina M. Crava

**Affiliations:** 1 Biotechnological Control of Pests Laboratory, Institute of Biotechnology and Biomedicine (BIOTECMED), Universitat de València, Burjassot, Valencia, 46100 Spain; 2 Plant Immunology and Biochemistry group, Department of Biology, Biochemistry and Environmental Sciences, Universitat Jaume I, Castellón de la Plana, 12071 Spain

**Keywords:** Beet armyworm, Egyptian cotton leafworm, Orally secreted proteins, Plant-insect interactions

## Abstract

The *Spodoptera* genus is defined as the pest-rich genus because it contains some of the most destructive lepidopteran crop pests, characterized by a wide host range. During feeding, the caterpillars release small amounts of oral secretion (OS) onto the wounded leaves. This secretion contains herbivore-induced molecular patterns (HAMPs) that activate the plant defense response, as well as effectors that may inhibit or diminish the plant’s anti-herbivory response. In this study, we explored the protein components of the OS of two *Spodoptera* species, *Spodoptera exigua* and *Spodoptera littoralis*. We identified 336 and 276 proteins, respectively, with a major role in digestion. Using a label-free quantitative proteomics approach, we investigated changes in protein abundance in the OS of both species after switching from a laboratory artificial diet to detached pepper and tomato leaves. Several proteins, such as various lipases, polycalin and a β-1,3-glucan binding protein, were more abundant in the OS of leaf-fed larvae in both species. Conversely, a tryptophan-aspartic acid (WD)-repeat containing protein significantly decreased upon feeding on plant leaves in both species. Phenotypic plasticity dependent on each *Spodoptera*-plant combination was observed for several peptidases, potentially related to the need to overcome the effects of proteinase inhibitors differentially produced by the two plant species, and for several REPAT proteins, possibly related to the specific modulation of each *Spodoptera*-plant interaction. Altogether, our results provide useful information for understanding the interaction of these two polyphagous *Spodoptera* species with the host plants, and help to identify evolutionary traits that may influence the outcome of herbivory in each of these two related species.

**Supplementary Information:**

The online version contains supplementary material available at 10.1007/s10886-025-01571-9.

## Introduction

Insect herbivory is a significant factor limiting plant growth and fitness. Consequently, plants have developed sophisticated strategies to perceive and counteract insect attack, including the production of direct defenses targeting insect nutrient absorption and development (Jones et al. 2022; Schuman and Baldwin 2016). The ability to induce anti-herbivore defenses allows plants to allocate energy to growth and reproduction when herbivory is absent, and to direct resources to defenses only when necessary. This trade-off enhances plant adaptability to biotic conditions and overall increases fitness (Karban and Baldwin 1997; Moreira et al. 2014).

When plants are attacked by herbivorous insects, they perceive molecules derived both from mechanical damage (damage-associated molecular patterns, DAMPs) (Acevedo et al. 2015; Tanaka and Heil 2021) and from the specific action of the insect (Acevedo et al. 2015; Jones et al. 2022). These latter compounds, known as herbivore-associated molecular patterns (HAMPs), indicate the presence and the nature of the attacker (Acevedo et al. 2015; Jones et al. 2022; Malik et al. 2021). HAMPs are present in all secretions produced by insects, such as saliva, regurgitate, frass, and even eggs (Jones et al. 2022; Zebelo et al. 2014). In the case of chewing caterpillars, many studies have focused on the characterization of the oral secretion (OS) deposited on leaves during feeding (Peiffer and Felton 2009). OS is a mixture of regurgitate and saliva, which contains molecules of different natures. Some of these molecules act as HAMPs, eliciting plant defense responses, while others may interfere with plant defense responses against herbivory, acting as effectors (Jones et al. 2022; Stam et al. 2014).

Among the molecules present in caterpillar’s OS and characterized as HAMPs are fattyacid–aminoacid conjugates (FACs) (Tumlinson and Lait 2005), such as the volicitin from *Spodoptera exigua* (Alborn et al. 1997), digestive enzymes like β-glucosidase (Mattiacci et al. 1995), and glucose oxidase (Eichenseer et al. 1999). This latter may also act as effector, suppressing plant defense response, and its role as elicitor or effector appears to be context-dependent (Jones et al. 2022; Shinde et al. 2024). Beside glucose oxidase, other molecules in the OS of caterpillars may decrease plant defenses (Acevedo et al. 2018, 2019; Chen et al. 2019, 2023; Liu et al. 2004; Shinde et al. 2024; Wu et al. 2012). For instance, *Helicoverpa armigera* R-like protein 1 (HARP1) (Chen et al. 2019) and Highly Accumulated Secretory Protein 1 (HAS1) (Chen et al. 2023) has been identified in *H. armigera*, and recently also in *Spodoptera frugiperda* (Zhang et al. 2024). HARP1 interacts with JAZ repressors, which are negative regulators of jasmonate defense response, protecting them from ubiquitination and degradation (Chen et al. 2019), while HAS1 inhibits the activation of transcription factors involved in defense compound biosynthesis (Chen et al. 2023). Both HARP1 and HAS1 belong to a large gene family of small proteins called REPAT (REsponse to PAThogens) (Herrero et al. 2007), which has at least 46 members in *S. exigua* (Navarro-Cerrillo et al. 2013). It is possible that other REPATs may be present in the caterpillar’s OS and they may decrease the host plant defense response by binding different activators and repressors of transcription involved in JA signaling, finally providing enhanced fitness to the herbivore.

Additionally, the composition of the caterpillar OS is not static but varies according to the plant diet, adapting to specific host plant species (Chen et al. 2019; Zhang et al. 2024; Zheng et al. 2022). For instance, diet-dependent changes have been thoroughly characterized in the saliva of many caterpillars, in particular the glucose oxidase activity (Acevedo et al. 2017; Afshar et al. 2010, 2013; Hu et al. 2008; Merkx-Jacques and Bede 2005; Peiffer and Felton 2005). These compositional changes are not limited to salivary secreted proteins but also involves changes in digestive enzymes, such as peptidases (Zhang et al. 2024; Zheng et al. 2022). This phenotypic plasticity may be related to need of polyphagous insects, which feed on a variety of plant species, to adapt to different host plants that may have diverse defenses and nutritional profiles.

Among polyphagous species, members of the Noctuidae family have been extensively studied because they are some of the most destructive lepidopteran crop pests (Kergoat et al. 2021). In particular, member of the genus *Spodoptera*, such as *S. exigua*, *Spodoptera littoralis*, and the invasive *S. frugiperda*, are models for the understanding of their relationships with crop host plants. Although the host plant repertoire of these three species largely overlaps, plant defense responses against these three attackers may not always be the same, and this may be potentially mediated by differences in OS composition. For instance, differential elicitation of plant defenses against S. *frugiperda* corn- and rice-strain seems to correlate with phospholipase C activity levels in the saliva (Acevedo et al. 2018). In this study, we use a comparative proteomics approach to characterize the OS of *S. exigua* and *S. littoralis* caterpillars when feeding on different plant substrates and artificial diet, with the aim of polarizing evolutionary traits between these two related species that share similar feeding behaviors.

## Materials and Methods

### Insects

*S. exigua* and *S. littoralis* colonies have been maintained for more than 100 generations at the University of Valencia (Spain). *S. exigua* colony was originally founded from eggs provided by Andermatt Biocontrol AG (Grossdietwil, Switzerland) whereas *S. littoralis* colony was founded with pupae provided by Primitivo Caballero Murillo’s laboratory (Instituto de Agrobiotecnología, CSIC, Navarra, Spain). Both species were fed with artificial diet (Elvira et al. 2010) when in larval stages and with *ad libitum* sugar solution (10% w/w) during adult stage. Rearing conditions for both species were the following: 25 ± 3 °C, 70 ± 5% of relative humidity and a photoperiod of 16 h light: 8 h dark.

### Plants

Tomato plants (*Solanum lycopersicum* var. Moneymaker) and pepper plants (*Capsicum annum* var. Dulce de España) were used in the experiment. The Moneymaker tomato is a variety widely used in *Spodoptera*-tomato interaction studies and exhibits a high tolerance to both *S. exigua* and *S. littoralis* (Frattini et al. 2022; Rivero et al. 2021; Ferrero et al. 2019). In contrast, The Dulce de España pepper shows lower tolerance to *Spodoptera* infestation compared to tomato, allowing greater caterpillar growth (data not shown). Both varieties were germinated and maintained in the greenhouse facility of the University of Valencia (Spain) until the experiment. The germination was achieved placing the seeds on Jiffy seedbeds and watered by flooding. Two weeks after germination, the seedlings were transplanted into pots with soil substrate and maintained with drip irrigation.

When the plants fully developed their fourth leaf (around 3–4 weeks from germination), they were transferred to a growth chamber under controlled conditions of 25 °C with 60% relative humidity and a photoperiod of 16 h light: 8 h dark, and used for rearing caterpillars until floral buds developed.

### Experimental Design and Oral Secretion Collection

Caterpillars used for oral secretion (OS) collection were reared on three different diets: artificial diet (Elvira et al. 2010), detached tomato leaves, and detached pepper leaves. In the case of the artificial diet and pepper leaves, the larvae were fed on these diets from hatching to the fifth instar. In contrast, for the tomato leaves, the larvae were fed with artificial diet from hatching to the second instar, and then the diet was switched to tomato leaves. This was due to the nearly 100% larval mortality that was observed when larvae were fed with tomato leaves since neonates. When larvae of both species reached the fifth instar, the OS was collected directly from the oral cavity using a 10 µL pipette while gently massaging the top of the larva’s heads. After collection, OS from 10 individuals was pooled together and immediately frozen at -80 °C until processing.

### SDS-PAGE and Qualitative Proteomics Analyses

To identify proteins present in the OS of the two caterpillar species, we created a peptide spectral library for each species combining samples from the three different rearing conditions (three biological replicates for artificial diet, tomato and pepper leaves). The proteomics analyses were carried out by the SCSIE facilities of the University of Valencia (Spain). The samples (20 µL each) were centrifuged at 21,000 x *g* at 4 °C for 10 min to remove any food debris and then cleaned by precipitation at 5 ºC with 20 µL of trichloroacetic acid (TCA). After precipitation, samples were centrifuged during 30 min at 21,000 x *g* at 5ºC. Pellets were then cleaned with 20 µL of cold acetone and centrifuged again as described above. The cleaning procedure was repeated 3 times. The precipitated protein pellets were resuspended in 20 µL of 1.5X Laemmli buffer (Bio-Rad) supplemented with 2-mercaptoethanol. Protein concentration in the sample was quantified with the Protein Quantification Assay kit (Macherey-Nagel), following the manufacturer’s instructions. After quantification, proteins from different replicates and treatments were pooled together (obtaining 27 µg of protein in total), denatured at 95ºC for 5 min and loaded in a single lane of an 12% Mini-PROTEAN TGX Precast Protein Gels (Bio-Rad). Gels were stained with colloidal blue and lanes were carefully cut in five different fragments. Proteins from each fragment were then digested in-gel using 1 µg of trypsin (Promega) as previously described (Shevchenko et al. 1996). The digestion was stopped by adding trifluoroacetic acid (TFA; 1% final concentration), and a double extraction with neat acetonitrile (ACN) was done. Both supernatants were combined and the final peptide solutions were dried in a rotatory evaporator and resuspended with 2% ACN; 0,1% TFA.

Five µL of peptide mixtures were initially loaded onto a trap column (3 µ C18-CL, 350 μm x 0.5 mm; Eksigent) and desalted with 0.1% TFA at 5 µL/min during 5 min. Subsequently, the peptides were resolved using an analytical column (3 µ C18-CL 120 Ᾰ, 0.075 × 150 mm; Eksigent) equilibrated in 5% ACN with 0.1% formic acid (FA). Chromatography was performed using a linear gradient 7–37% of buffer B (ACN and 0.1% FA) in buffer A (0.1% FA) at a flow rate of 300 nL/min during 60 min. Eluted peptides were analysed by mass spectrometry using a nanoESI qQTOF 6600plus TripleTOF (AB Sciex). Samples were ionized in a Optiflow ion source applying 3.0 kV to the spray emitter at 200 ºC. The instrument was operated in data-dependent acquisition mode. Survey MS1 scans were acquired from 350 to 1400 m/z for 250 ms. The quadrupole resolution was set to ‘LOW’ for MS2 experiments, which were acquired from 100 to 1500 m/z for 25 ms in ‘high sensitivity’ mode. Following switch criteria were used: charge: 2 + to 4+; minimum intensity; 250 counts per second (cps). Up to 100 ions were selected for fragmentation after each survey scan. Dynamic exclusion was set to 15 s. The rolling collision energies equations were set according to the following equations: *|CE|=(slope)x(m/z) + (intercept) for all ions as for + 2 (Slope = 0*,*049; Intercept = 2)*.

Proteins were identified using the Paragon algorithm (Shilov et al. 2007) in ProteinPilot v 5.0 (AB Sciex), combining all the information produced by the processing of the five gel fragments. The query parameters were trypsin specificity and iodoacetamide cys-alkylation. To construct the proteome, we performed protein identification searches against Uniprot libraries restricted to Lepidopteran, and contaminants from diet (wheat, corn and solanaceous plants). Only proteins with a global false discovery rate (FDR) < 1% (> 95% confidence by the Paragon algorithm’s internal scoring metrics) (Sennels et al. 2009), which corresponded to Protein Pilot Unused Score > 1.3, were considered to be confidently identified. The Protein Pilot Unused Score is based on the Unused Score’s concept, which represents the contribution of a unique peptide to the identification of a protein. When multiple unique peptides are identified for the same protein, the Unused Scores of these peptides are combined to generate a global score for that protein. This global score reflects the relative abundance of the protein in the sample, as more abundant proteins tend to have more identified peptides with higher values. Therefore, the highest Unused Score values were selected to compose the top ten most abundant proteins in the OS of each *Spodoptera* species.

Functional annotation of proteins identified in the oral secretion of *S. exigua* and *S. littoralis* was run using the Blast2GO pipeline (Götz et al. 2008) implemented in OmicsBox v3.1.11. Briefly, a fasta file containing all proteins identified by Protein Pilot was downloaded from UniProt, and these were used as queries in blastp and InterProScan searches with Blast2GO default parameters. GO terms were then merged, mapped and annotated, and enzyme codes (ECs) were assigned. Lollipop plots are created using the dplyr (Wickham et al. 2023) and ggplot2 (Wickham 2016) packages in R v4.3.1 (R Core Team 2018).

### Quantitative Proteomics Analyses

Samples precipitated with TCA as described above were used for SWATH analysis. Briefly, ten µg of every sample was digested following SP3 protocol (Hughes et al. 2019) and using 400 ng of trypsin at 37ºC. Digested peptides were acidified with 10% TFA to a final concentration of 1%. Peptides mixtures were dried in a rotatory evaporator and dissolved with 10 µL of 0.1% TFA in 2% ACN. Three µL of each peptide mixture sample were resolved in an Ekspert nanoLC-425 (Eksigent) liquid chromatography system coupled in mass spectrometry analysis. Samples were initially desalted with 0.1% TFA at 5 µL/min during 3 min on a trap column (3µ C18-CL, 350 μm x 0,5 mm; Eksigent) and then eluted onto a 15 cm analytical column (3µ C18-CL 120 Ᾰ; Eksigent) equilibrated in 7% acetonitrile and 0.1% FA. Chromatography was performed using a linear gradient 7–37% of buffer B (ACN and 0.1% FA) in A (0.1% FA) at a flow rate of 300 nL/min during 120 min. Peptides were analysed in a mass spectrometer nanoESI qQTOF (6600plus TripleTOF, AB Sciex). Samples were ionized in a Optiflow ion source applying 3.0 kV to the spray emitter at 175 ºC. The instrument was operated in SWATH mode, in which a 50 ms TOF MS scan from 350 to 1250 m/z was performed. After, 80 ms product ion scans in 100 variable windows from 400 to 1250 m/z were acquired throughout the experiment. All the ions were fragmented as + 2 ions. The total cycle time was 2.79 s. The individual SWATH injections were randomized to avoid bias in the analysis.

The wiff files obtained from SWATH experiment were analyzed by DIA-NN v1.8 (Demichev et al. 2020) to obtain label-free quantification (LFQ) values using an in silico-predicted spectral library generated from the spectral peptide library produced during the qualitative proteomics analysis. The DIA raw data files were then analyzed with the following parameters: automatic inference of mass accuracy, MS1 accuracy, and scan window; removal of likely interferences; neural network classifier, double pass mode; quantification strategy; any LC (high accuracy); cross-run normalization, RT-dependent; library generation, Smart profiling; speed and RAM usage, optimal results. At the end of the analysis, a matrix file was obtained which contained normalized LFQ values for protein groups that were filtered at 1% FDR, using global q-values for protein groups and both global and run-specific q-values for precursors. Data are available via ProteomeXchange with identifier PXD056472.

Statistical analysis of multivariate quantitative protein abundances was performed within the Perseus software platform (Tyanova et al. 2016). LFQ values produced by DIAN-NN were initially filtered for data present in the 70% of the replicates of at least one treatment. Later, variables with missing values were imputed by random draws from independent normal distributions. Data were then normalized using Z-scores and differences in abundances among all groups were analyzed using ANOVA. Principal component analysis (PCA) was then conducted on statistical different proteins to confirm the clustering of the treatments. Finally, pairwise-comparisons between specific treatments were performed using Student t-test. A protein abundance was considered significant different when it had a p-value lower than 0.05 and fold-change (FC) higher than 2. Volcano plots to were created with R version 4.3.1 packages ggplot2 (Wickham 2016) and EnhancedVolcano (Blighe et al. 2018).

## Results

### Proteins Identified in the Oral Secretion of *S. Exigua* and *S. Littoralis*

In the present study, we generated 98,374 and 87,329 MS/MS spectra for *S. exigua* and *S. littoralis* oral secretion (OS), respectively. In *S. exigua*, 40,274 unique spectra were assigned to 7,965 distinct peptides, leading to the identification of 661 different proteins at > 95% confidence. After removing the proteins associated with diet, there were 336 different proteins identified at a high confidence level (Supplementary Table 1). In *S. littoralis*, 19,404 unique spectra were assigned to 4,516 distinct peptides, resulting in the identification of in 455 proteins at > 95% confidence. After removing the proteins associated with diet, 276 proteins were identified at a high confidence level (Supplementary Table 2).

**Fig. 1:**
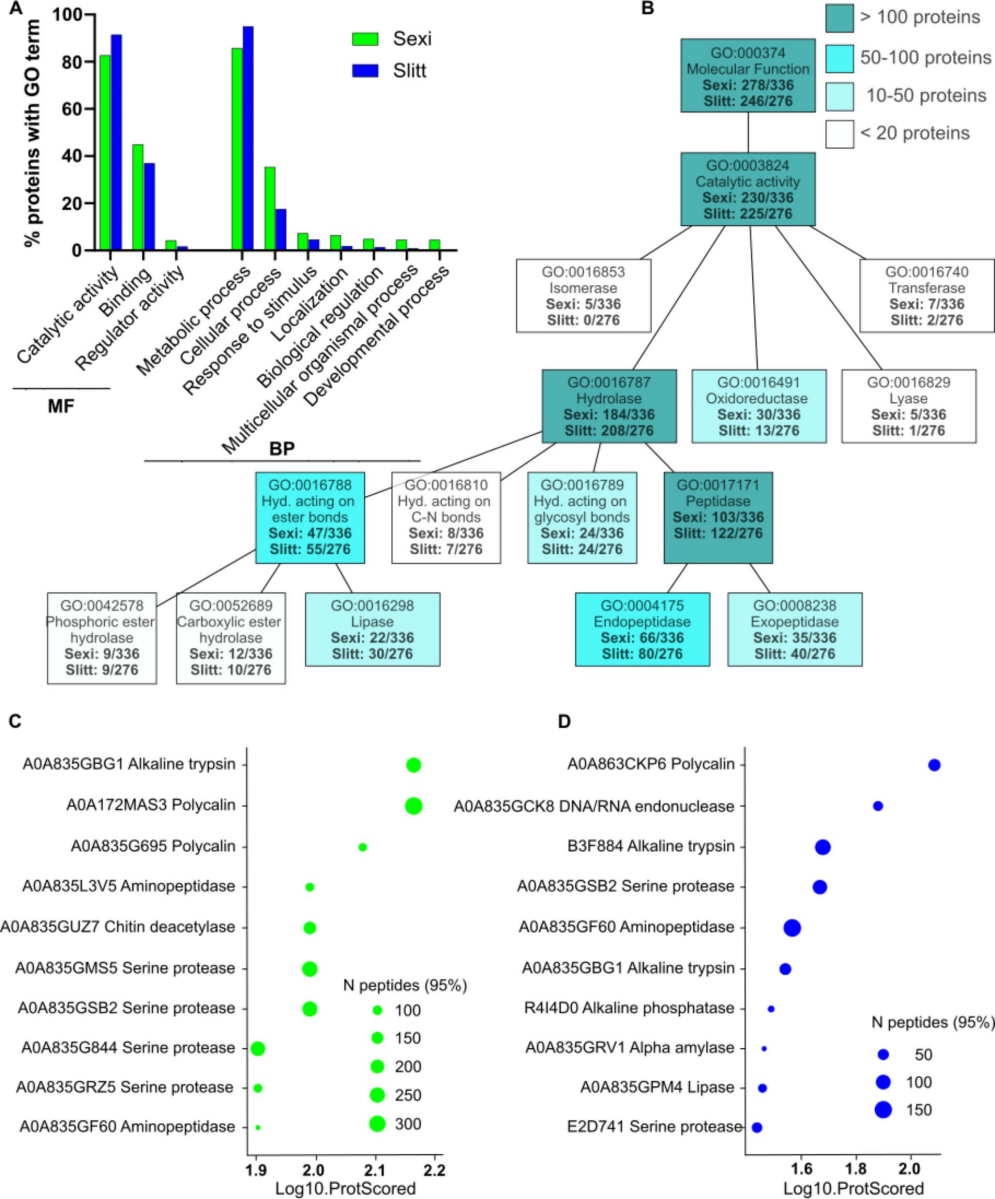
Qualitative proteomics analysis of oral secretion from *S. exigua* and *S. littoralis*. **(A**) Gene Ontology (GO) term analysis using the program Blast2GO identified ten “level 2” terms with at least 10 proteins in at least one species. Abbreviations: MF, Molecular Function; BP, Biological Process. **(B) **Lower-level Molecular Function GO terms highlighting the number or proteins with catalytic activity in the oral secretion of both species. Lower-level terms were selected to illustrate most abundant functions in the datasets. Abbreviation: hyd., hydrolase. **(C)** Lollipop plot of top ten most abundant proteins in the oral secretion of *S. exigua *(left panel) and *S. littoralis* (right panel). Uniprot accession number and description of the best blastp hit against nr (NCBI non-redundant protein sequence)database are reported

To identify proteins involved in specific functions, we examined their annotations with Gene Ontology (GO) terms using Blast2GO, which combines both Blast and Uniprot searches. GO terms refer to cellular components, biological processes, or molecular functions with which a protein is associated. Of the 336 proteins in the oral secretion of *S. exigua*, 278 were assigned a molecular function (Fig. 1A). Among these, a large array of proteins exhibited catalytic functions (230), with 184 proteins described by the hydrolase activity term, and an additional 30 proteins described by the oxidoreductase activity term (Fig. 1B). Of the proteins described with hydrolase activity, 47 were identified as acting on ester bonds (of these, 22 as lipase activity), 24 acting on glycosyl bonds, while the majority (103) were described as peptidase activity. Of these, 66 proteins were assigned endopeptidase activity and 35 proteins, exopeptidase activity. A smaller number of proteins (11) were assigned to peptidase inhibitor activity, and hence possibly involved in the regulation of the proteolysis. A similar scenario was observed for *S. littoralis* where 246 out of 276 proteins were assigned a molecular function (Fig. 1A). Among these, the large majority (225) had catalytic activity, with 13 proteins having oxidoreductase activity and 208 proteins having hydrolase activity. These were divided into 24 proteins acting on glycosyl bonds, 55 proteins acting on ester bonds (of which 30 had lipase activity), and 122 with peptidase activity (80 with endopeptidase and 40 with exopeptidase activity) (Fig. 1B). In *S. littoralis*, proteins with a term as peptidase inhibitor activity were four.

In terms of biological process, in *S. exigua*, 247 proteins were assigned a GO term with 212 involved in metabolic processes (Fig. 1A). Specifically, 29 proteins assigned to carbohydrate metabolic process, 21 to amide metabolic process, 37 to lipid metabolic process, 25 to organic cyclic compound metabolic process and 113 to protein metabolic process. Within the latter category, 106 proteins were dedicated to proteolysis, constituting the 29% of the total count of identified proteins. In *S. littoralis*, out of the 201 proteins assigned a biological process GO term, 194 were involved in metabolic process (Fig. 1A). Among these, 24 proteins dedicated to carbohydrate metabolic process, 37 to lipid metabolic process, 10 to organic cyclic compound metabolic process and 116 to protein metabolic process. Of the latter, 113 proteins were involved in proteolysis, representing the 41% of the total count of identified proteins.

Examining the top ten most abundant proteins in the oral secretion of *S. exigua* (Fig. 1C) and *S. littoralis* (Fig. 1D), the presence of proteases was prominent in both species. Specifically, seven proteases were found in *S. exigua* and five in *S. littoralis*, with two endopeptidases and one exopeptidase being identical between the two species. Interestingly, aside from the proteins likely involved in digestion, polycalin stands out in both species. In *S. exigua*, two polycalins were among the most abundant proteins in the OS, while one polycalin was found in *S. littoralis* OS. Polycalin is a lipocalin specialized in binding chlorophyllide A and is specifically expressed in the midgut (Mauchamp et al. 2006). Its presence in the oral secretion may be related to the need to immediately bind chlorophyllide present in the plant diet.

### Differential abundance profiles of proteins contained in the oral secretion of *S. exigua* larvae raised on different diets

**Fig. 2:**
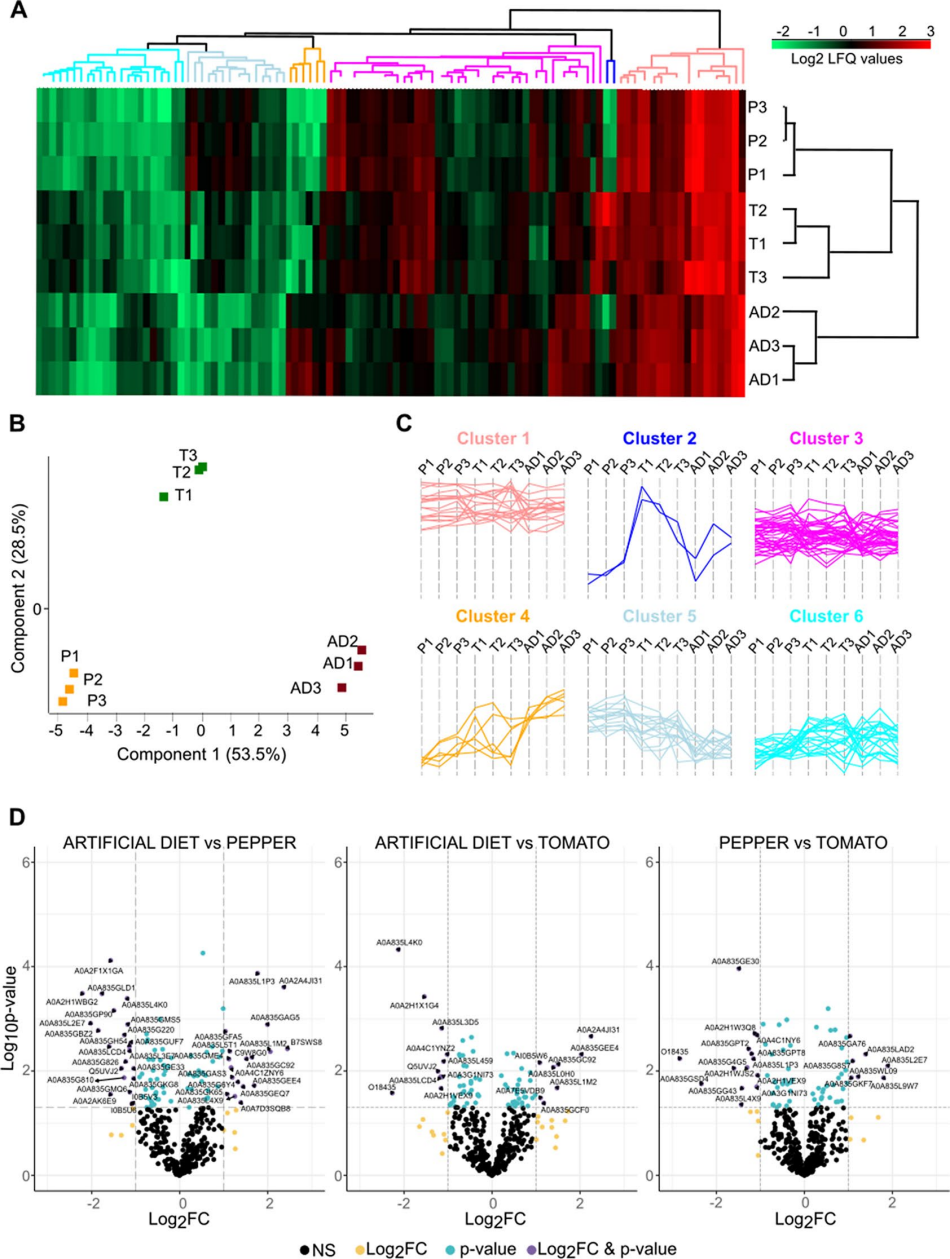
Differentially abundant proteins in the oral secretion of *S. exigua*. (**A**) Heatmap illustrating Z-score normalized label-free quantification (LFQ) DIA-NN values of the 105 proteins whose abundances were statistically different among treatments according to ANOVA (p value < 0.05). Protein clusters in the dendrogram are color-coded according to panel C. (**B**) Principal component analysis (PCA) of Z-score normalized LFQ DIA-NN values of proteins whose abundances were statistically different among treatments according to ANOVA (p value < 0.05). P1, P2, and P3 are the OS samples (biological replicates) from larvae fed on detached pepper leaves; T1, T2, and T3 are the OS samples (biological replicates) from larvae fed on detached tomato leaves; AD1, AD2, and AD3 are the OS samples (biological replicates) on larvae fed an artificial diet. (**C**) Profile plots of six clusters showing distinct profiles with respect to diet. (**D**) Volcano plots displaying the Uniprot ID of the proteins with significantly altered abundance according to pairwise Student’s t-test (*p* < 0.05 and log2 fold-change, FC). NS, non-significant

The abundance profiles of proteins in the OS of *S. exigua* larvae under different diet treatments (artificial diet–AD-, detached pepper leaves, and detached tomato leaves) were analyzed using a label-free quantification (LFQ) approach. A total of 105 proteins showed significant differences in abundance in at least one treatment, as determined by ANOVA (Fig. 2A, Supplementary Table 3). Principal component analysis (PCA) (Fig. 2B) and hierarchical clustering (Fig. 2A) of these differentially abundant proteins clearly separated the three diet groups. Hierarchical clustering identified six distinct clusters with different patterns of protein abundance (Fig. 2C). Among these, three clusters exhibited specific signatures associated with particular diet treatments. Cluster 2 grouped two proteins with higher abundance in larvae fed on tomato compared to the other two treatments, Cluster 4 contained proteins with higher abundance in larvae fed on AD, and Cluster 5 included proteins with higher abundance in larvae fed on pepper (Fig. 2C, Supplementary Table 3).

**Fig. 3:**
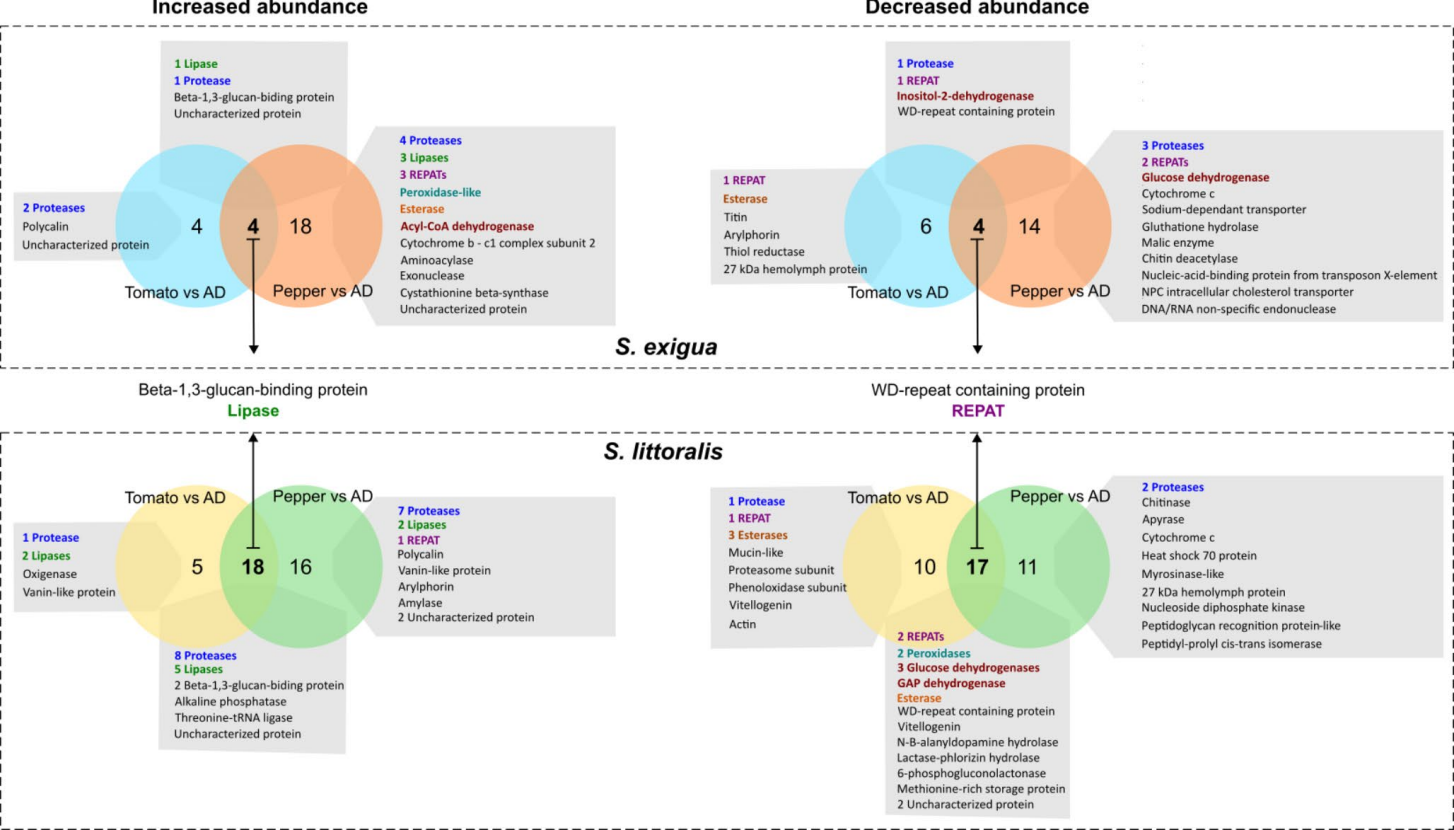
Comparison of differentially abundant proteins according to diet in *S. exigua* and *S. littoralis*. Venn diagrams illustrated the number of differentially abundant proteins in the oral secretion (OS) of *S. exigua* (upper panels) and *S. littoralis* (lower panels) based on the diet they were fed (AD: artificial diet). Next to the Venn diagrams, the descriptors of the best blastp hits for the differentially abundant proteins are displayed. Arrows indicate the proteins that showed differential abundance in both plant-based diet treatments vs. AD and are common to both species. Different classes of proteins are highlighted with different colors (proteases, blue; REPATs, purple; dehydrogenases, red; esterases, orange; lipases, green; petrol blue, peroxidases)

We next explored the pairwise differences triggered by the different diet treatments to identify *S. exigua* OS proteins whose abundances significantly change by at least twofold in any specific contrast. When *S. exigua* caterpillars fed on detached leaves compared to those on AD, we observed changes in the abundance of 18 proteins in tomato-fed larvae and 40 proteins in pepper-fed larvae (Fig. 2D). Among these, we identified eight proteins that displayed consistent changes in abundances concordantly across both plant treatments (Fig. 3). Specifically, the OS of *S. exigua* larvae reared on plant leaves contained four proteins with significantly higher abundance and four with significantly lower abundance compared to larvae raised on AD (Table 1). Proteins with increased abundance in the OS of leaf-fed larvae were a β-1,3-glucan binding protein, an aminopeptidase N, a lipase and an uncharacterized protein. In contrast, a REPAT-like protein, a trypsin, an inositol 2-dehydrogenase and a tryptophan-aspartic acid (WD) repeat-containing protein were less abundant in the OS of leaf-fed larvae. Interestingly the WD-repeat containing protein was among the proteins with the lowest abundance in both contrasts (5.2 and 4.7 FC in pepper vs. AD and tomato vs. AD comparisons, respectively) (Table 1).

Among the 32 proteins whose abundances significantly changed only in pepper-fed larvae compared to AD-fed larvae, 18 showed increased abundance (Fig. 2D). Among these were four peptidases, three lipases, and three REPATs (Fig. 3). The largest increase in abundance was observed in a peroxidase (4.6 fold-change, FC), followed by REPAT23 (4.1 FC) (Supplementary Table 4). On the other side, among the 14 proteins with decreased abundance, we identified three peptidases and two REPATs, as well as a two proteins glucose dehydrogenase (Fig. 3). The protein with the greatest reduction in abundance was REPAT2 (5.5 FC) (Supplementary Table 4). Interestingly, the decrease in abundance of two trypsins, one REPAT, and a transposable nucleic-acid-binding protein in the OS of pepper-fed larvae compared to AD-fed larvae was also statistically significant in the contrast between pepper-fed larvae and tomato-fed larvae, emphasizing their potential specific role in the interaction between *S. exigua* and pepper. Likewise, REPAT23 was statistically more abundant in the OS of pepper-fed larvae compared to both AD-fed and tomato-fed larvae (Supplementary Table 4).

In the OS of tomato-fed larvae compared to AD-fed larvae there were 10 proteins that changed their abundances only in this contrast.Specifically, four proteins showed increased abundance and si decreased abundance (Fig. 3). Among the four proteins with increased abundance there were two peptidases and the polycalin. Notably, one of the peptidases showed a 4.8-fold increase in abundance in tomato fed-larvae compared to AD-fed, and a 7.1-fold increase compared to pepper-fed larvae (Supplementary Table 4). Among the six proteins whit decreased abundance in tomato-fed larvae compared to AD-fed larvae, were an esterase, a REPAT, titin, and two hemolymph proteins (arylphorin and 27-kDa hemolymph protein) (Fig. 3). Arylphorin and titin also showed a significant decrease in the comparison between tomato-fed and pepper-fed larvae.

Lastly, the comparison of protein abundance in the OS of pepper-fed versus tomato-fed larvae also revealed additional changes (Fig. 2D). Thirteen proteins showed significant changes between the two leaf-based diets but not in comparison to AD (Supplementary Table 4). Specifically, seven proteins were most abundant in the OS of pepper-fed larvae, while six were more abundant in tomato-fed larvae. The latter group included two REPATs, two peptidases, and a lipase (Supplementary Table 4).

**Table 1:** Statistically significant fold-changes (absolute FC > 2 and p value < 0.05) in abundance of *S. exigua* OS proteins identified in both pairwise contrasts between leaf-fed (pepper and tomato) and AD-fed larvae

Uniprot ID	Best Blastp hit descriptor	Pairwise comparisons		
		Pepper-AD^a^ fold-change	Tomato-AD^a^ fold-change	Tomato-pepper fold-change
A0A835LCD4	Lipase	3.0	2.3	ns^a^
A0A2H1X1G4	Uncharacterized protein	3.0	2.9	ns^a^
Q5UVJ2	Aminopeptidase N	2.5	2.3	ns^a^
A0A835L4K0	Beta-1,3-glucan-binding protein	2.3	4.4	ns^a^
A0A2A4JI31	WD repeat-containing protein	-5.2	-4.7	ns^a^
A0A835L1M2	REPAT-like	-4.2	-2.8	ns^a^
A0A835GEE4	Trypsin	-3.2	-4.1	ns^a^
A0A835GC92	Inositol 2-dehydrogenase	-2.9	-2.8	ns^a^

^a^ Abbreviations: AD, artificial diet; ns, non-significant

### Differential abundance profiles of proteins contained in the oral secretion of *S. littoralis* larvae raised on different diets

**Fig. 4:**
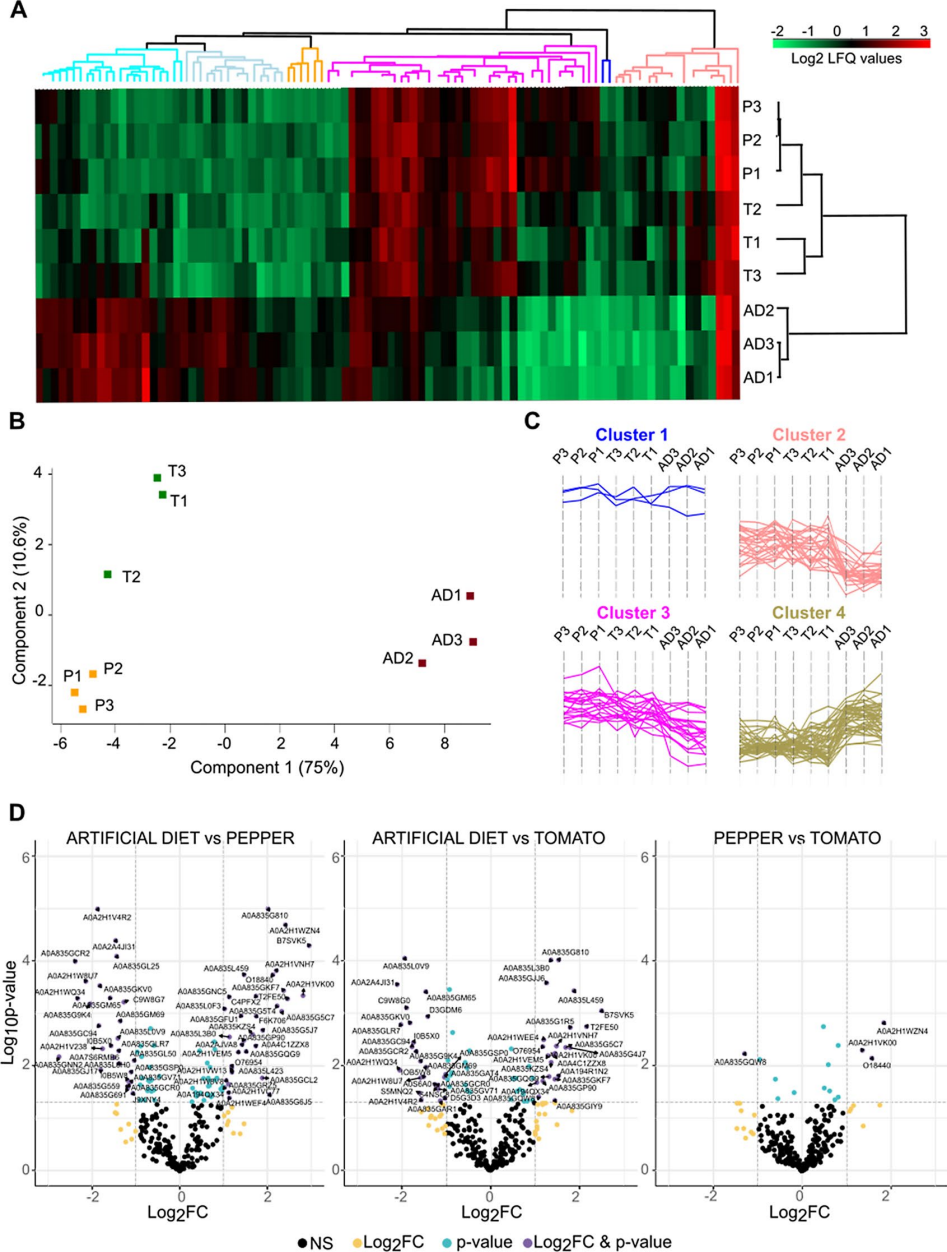
Differentially abundant proteins in the oral secretion of *S. littoralis*. (**A**) Heatmap illustrating Z-score normalized label-free quantification (LFQ) DIA-NN values of the 105 proteins whose abundances were statistically different among treatments according to ANOVA (p value < 0.05). Protein clusters in the dendrogram are color-coded according to panel C. (**B**) Principal component analysis (PCA) of Z-score normalized LFQ DIA-NN values of proteins whose abundances were statistically different among treatments according to ANOVA (p value < 0.05). P1, P2, and P3 are the OS samples (biological replicates) from larvae fed on detached pepper leaves; T1, T2, and T3 are the OS samples (biological replicates) from larvae fed on detached tomato leaves; AD1, AD2, and AD3 are the OS samples (biological replicates) on larvae fed an artificial diet. (**C**) Profile plots of six clusters showing distinct profiles with respect to diet. (**D**) Volcano plots displaying the Uniprot ID of the proteins with significantly altered abundance according to pairwise Student’s t-test (*p* < 0.05 and log2 fold-change, FC). NS, non-significant

Using the same LFQ quantification method as for *S. exigua*, we identified 92 proteins from the OS of *S. littoralis* caterpillars that showed differential abundance profiles according to the diet on which the larvae were fed, as determined by ANOVA (Fig. 4A, Supplementary Table 5). Principal component analysis (PCA) of these differentially abundant proteins clearly distinguished between plant diet-based diet treatments and AD, although the replicates for the tomato and pepper treatments clustered more closely together (Fig. 4B) than in *S. exigua* (Fig. 2B). Consistently, hierarchical clustering of the differentially abundant proteins resulted in a tomato sample being included in the pepper cluster (Fig. 4A). Four clusters representing different patterns of differential abundance were identified (Fig. 4C, Supplementary Table 5), with three of these clusters showing specific signatures linked to particular diet treatments. Unlike in *S. exigua*, the differences in OS protein abundances in *S. littoralis* were mainly driven by the comparison between plant-based diets and AD. Specifically, Clusters 2 and 3 grouped proteins with higher abundance in the OS of larvae fed on either plant diets compared to AD, while Cluster 4 grouped proteins with higher abundance in OS of larvae fed AD compared to plant diets (Fig. 4C).

Pairwise comparisons between treatments revealed that eighteen *S. littoralis* OS proteins showed an increased abundance in both plant-based diet groups compared to AD- fed larvae (Fig. 3). Similarly, seventeen proteins showed decreased abundance in the OS of both pepper-fed larvae and tomato-fed larvae compared to AD. Among proteins with increased abundances in both leaf-fed groups, we identified two β-1,3-glucan binding proteins, one of which exhibited the highest abundance increase in both comparisons (7.7 and 5.7 FC in pepper-AD and tomato-AD contrasts, respectively) (Table 2). Other proteins that were more abundant in the OS of both leaf-fed larvae were five lipases and eight peptidases (namely, three exopeptidases and five endopeptidases). Conversely, among the proteins whose abundance decreased, we identified an esterase, two peroxidases, two REPATs, a WD-repeat containing protein and four proteins potentially involved in energy metabolism (three glucose dehydrogenases and a glyceraldehyde-3-phosphate dehydrogenase) (Table 2).

Focusing on proteins with a differential abundance profile unique to the pepper-fed vs. AD-fed larvae comparison, sixteen proteins displayed increased abundance in pepper-fed larvae, including seven peptidases and two lipases (Fig. 3). Two of these, a lipase and a trypsin, also showed increased abundance in the OS of pepper-fed larvae compared to tomato-fed larvae. Additionally, we identified a REPAT, a polycalin and a vanin-like proteins. Conversely, eleven proteins showed decrease abundance in the OS of pepper-fed larvae compared to the AD diet, including two proteases and a chitinase, with the chitinase exhibiting the greatest reduction (-6.8 FC) (Supplementary Table 6).

In the contrast between tomato-fed and AD-fed larvae, five proteins displayed significant increased abundance only in this comparison. These included two lipases, an oxygenase, an aminopeptidase and a vanin-like protein (Fig. 3). One lipase also showed increased abundance in the OS of tomato-fed larvae compared to pepper-fed larvae. The OS proteins that showed a decreased abundance in tomato-fed compared to AD-fed larvae included ten proteins, among which were three esterases, a peptidase (collagenase) and a REPAT (Supplementary Table 6). No differences unique to the tomato-fed vs. pepper-fed larvae were identified in *S. littoralis* (Fig. 4D, Supplementary Table 5), likely due to the lack of clear separation between the tomato and pepper replicates (Fig. 4).

**Table 2:** Statistically significant fold-changes (absolute FC > 2 and p value < 0.05) in abundance of *S. littoralis* OS proteins identified in both pairwise contrasts between leaf-fed (pepper and tomato) and AD-fed larvae

Uniprot ID	Best Blastp hit descriptor	Pairwise comparisons		
		Pepper-AD^a^ fold-change	Tomato-AD^a^ fold-change	Tomato-pepper fold-change
B7SVK5	Beta-1,3-glucan-binding protein	7.7	5.7	ns^a^
A0A2H1VK00	Lipase	7.0	2.7	2.6
T2FE50	Beta-1,3-glucan-binding protein	5.5	4.5	ns^a^
A0A835GKF7	Carboxypeptidase B	5.2	2.7	ns^a^
A0A835G5C7	Lipase	5.0	3.2	ns^a^
A0A2H1VNH7	Carboxypeptidase B	4.6	2.8	ns^a^
A0A835G810	Lipase	4.0	2.9	ns^a^
A0A835G4J7	Lipase	3.7	3.5	ns^a^
A0A4C1ZZX8	Uncharacterized protein	3.3	2.7	ns^a^
A0A835KZS4	Peptidase	3.2	2.6	ns^a^
A0A835GQG9	Peptidase	2.9	2.5	ns^a^
A0A835L459	Carboxypeptidase B	2.8	3.7	ns^a^
A0A835GP90	Trypsin	2.7	2.3	ns^a^
A0A2H1VEM5	Lipase	2.5	2.4	ns^a^
O76954	Trypsin	2.3	2.3	ns^a^
A0A835L3B0	Alkaline phosphatase	2.2	2.6	ns^a^
A0A2H1WEE4	Trypsin	2.2	2.3	ns^a^
A0A194QX34	Threonine-tRNA ligase	2.0	2.1	ns^a^
A0A835GCR2	Esterase	-5.2	-3.2	ns^a^
A0A2H1WQ34	Glucose dehydrogenase	-5.0	-4.1	ns^a^
A0A2H1W8U7	Peroxidase	-4.4	-3.0	ns^a^
A0A835G9K4	N-β-alanyldopamine hydrolase	-4.1	-3.0	ns^a^
A0A2H1V4R2	Glucose dehydrogenase	-3.7	-2.9	ns^a^
A0A835GC94	Vitellogenin	-3.6	-3.4	ns^a^
A0A835GKV0	Uncharacterized protein	-3.5	-3.5	ns^a^
A0A835GM65	Uncharacterized protein	-3.0	-2.7	ns^a^
I0B5X0	REPAT43	-3.0	-3.3	ns^a^
A0A2A4JI31	WD repeat-containing protein	-2.8	-4.3	ns^a^
A0A835L0V9	Glucose dehydrogenase	-2.7	-3.8	ns^a^
A0A835GLR7	Lactase-phlorizin hydrolase	-2.6	-4.0	ns^a^
A0A835GM69	Peroxidase	-2.6	-2.7	ns^a^
I0B5W8	REPAT46	-2.3	-2.8	ns^a^
A0A835GCR0	6-phosphogluconolactonase	-2.2	-2.3	ns^a^
A0A835GV71	Glyceraldehyde-3-phosphate dehydrogenase	-2.2	-2.3	ns^a^
A0A835GSP0	Methionine-rich storage protein	-2.2	-2.0	ns^a^

^a^ Abbreviations: AD, artificial diet; ns, non-significant

## Discussion

In this study, we performed comparative proteomics analyses to explore how the secreted proteins present in the oral secretion of two generalist caterpillars from the *Spodoptera* genus, *S. exigua* and *S. littoralis*, are affected by different feeding substrates (artificial diet–AD-, detached tomato leaves, and detached pepper leaves), focusing on the conservation of these changes across the two species. This comparison allowed us to identify four proteins whose abundance increases or decreases consistently in both species when the caterpillars feed on either plant-based diet (tomato or pepper) compared to AD. These proteins may play a role in the interaction with plants, either in the digestive process or in triggering anti-herbivore defenses in the plant hosts. Additionally, we identified a larger number of proteins whose changes in abundance were specific to particular comparisons (i.e., in a single plant diet compared to AD, or in a single species). Although our functional interpretation of the data is limited due to the scarcity of functionally characterized *Spodoptera* proteins - particularly in *S. littoralis*, we relied heavily on gene ontology inferences based on annotated proteins from other species with similar protein sequences - weeare able to provide a comprehensive overview into the proteomic landscape of Spodoptera caterpillar oral secretions.

Overall, our results show that both species had fewer proteins with altered abundance (either increased or decreased) in the comparison between tomato-fed and AD larvae than in the comparison between pepper-fed and AD-fed larvae. This may be related to a higher tolerance of pepper than tomato, since our laboratory colonies, which have been maintained on an artificial diet for years, are unable to develop on tomato leaves from the first instar, whereas they can develop on pepper leaves. In nature, caterpillars of both *Spodoptera* species are generalists, and in the Mediterranean basin are important pest of pepper cultivation in greenhouses, occasionally feeding on tomato (Aparicio et al. 1998; García & Ferragut, 2002). Therefore, the difference in the number of differentially abundant orally secreted proteins may be reflect the varying tolerance of these two solanaceous crops. A striking distinction between *S. exigua* and *S.littoralis* is that, in the latter, the abundance profiles of orally secreted proteins in tomato-fed and pepper-fed larvae compared to AD-fed largely overlap. In contrast, in *S. exigua*, there is a much clearer separation between the proteins with altered abundance in the two leaf-based diets. This distinction was further emphasized by the groups identified through hierarchical clustering. Of the three clusters with distinct signatures in *S. exigua*, two group proteins with higher abundance in pepper- or tomato-fed larvae compared to all the other treatments, while the third groups proteins with higher abundance in AD-fed larvae. In contrast, *S. littoralis* showed clusters with clear signatures only in relation to changes between AD-fed larvae and those fed both leaves-based diets.

Our results show that more than 60% of the proteins in the oral secretion of *S. exigua* and 80% in *S. littoralis* are involved in metabolic processes. This is consistent with previous research showing that a significant proportion of oral secretion proteins from lepidopteran larvae are digestive enzymes (Chen et al. 2019; Zhang et al. 2024; Zheng et al. 2022). In both species, peptidases are the most abundant (around 30–40%), and lipases account for approximately 10%. Insect herbivores utilize a wide variety of peptidases with various specificities (such as trypsins, chymotrypsins, elastases, cathepsin-B like proteases, aminopeptidases and carboxypeptidase, among others) to cleave peptide bonds in plant proteins, both for primary protein digestion and to hydrolyze plant inhibitors. In Lepidoptera larvae, serine proteases are the most common type (Srinivasan et al. 2006). As an adaptive strategy, insects accumulate proteases that are not targeted by plant-derived protease inhibitors (Zhu-Salzman and Zeng 2015). We observed changes in peptidase abundances in both species when comparing leaf-fed vs. AD-fed larvae. This may be attributed to the larvae’s need to switch to different sets of proteases when feeding on various host plants that primarily use protease inhibitors as a defense mechanism, such as tomato and pepper (Rodriguez-Saona, 2010; Mishra et al. 2010). Globally, we observed a major increase in abundance of proteases when both *S. exigua* and *S. littoralis* switched from AD to leaf-based diet: 16 different types of proteases increased their abundances in *S. littoralis* when fed on a leaf-based diet, whereas only three decreased their abundances. A similar pattern was observed in *S. exigua*, where seven types of proteases showed increased abundances, while four showed decreases. Notably, also the switch from a tomato-based diet to a pepper-based diet changed the abundance of two proteases. However, this change was not detected in *S. littoralis*, likely due to the smaller differences between tomato and pepper replicates in this species.

Lipases are serine hydrolases defined as triacylglycerol acylhydrolases. They are responsible for the digestion of dietary fats, hydrolysis of lipids, and lipid mobilization (Lim et al. 2022). It has been observed that their activity in the oral secretion of the grasshopper *Schistocerca gregaria* can directly induce changes in the oxylipin 12-oxo-phytodienoic acid (OPDA), a biosynthetic precursor of the jasmonic acid, leading to its accumulation in Arabidopsis (Schäfer et al. 2011). Our findings show an increased abundance of several lipases when both *S. exigua* and *S. littoralis* larvae feed on both tomato and pepper leaves instead of artificial diet. This common pattern, which agrees with previous findings in another generalist *Spodoptera* species, *S. frugiperda* (Acevedo et al. 2018; Zhang et al. 2024), may be related to the needing to digest plant lipids. However, lipases seem to be elicitor of defenses not only in grasshoppers but also in *S. frugiperda*, since maize defense induction with OS mixed with a lipase inhibitor decreased the activation of defense responsive genes (Zhang et al. 2024) and differences in defense activation between the corn and rice strains were related to differences in the activity levels of a salivary phospholipase C (Acevedo et al. 2018). Hence, lipases present in leaf-fed caterpillars' OS may play a similar role in shaping plant-*S. exigua* or *-S. littoralis* interaction, acting as elicitors of plant defense.

Among other enzymes present in the OS, we found a general decrease of the abundance of esterases in the OS of both *Spodoptera* species when feeding on detached leaves (the abundance of three esterases in *S. littoralis* and one in *S. exigua* decreased, whereas one in *S. exigua* showed increased abundance). Esterases are Phase I detoxification enzymes which catalyze the hydrolysis of carboxylic acid esters, and have been related to the detoxification of plant defenses and pesticides (Zunjarrao et al. 2020). However, a reduction in the OS of leaf-fed larvae may be not correlated with the need to detoxify tomato and pepper defenses, in contrast to what observed in *S. frugiperda* feeding on maize (Zhang et al. 2024). A similar erratic pattern of abundance was shown by peroxidases. Peroxidases reduced reactive oxygen species (ROS), and may aid in deactivating ingested phytochemicals. In the mirid *Apolygus lucorum*, the peroxidase AI6 in an effector that suppresses ROS induced involved in the pattern-triggered immunity-induced cell death (Dong et al. 2020). In our study, *S. littoralis*’ OS displayed a decreased abundance of two peroxidases when feeding on both plants compared to artificial diet; however, in *S. exigua* a peroxidase was the protein with the greatest abundance increase in the OS of pepper-fed larvae. This last is consistent with *S. frugiperda*, where the abundance of a peroxidase increased in the OS of larvae that fed on three different plant substrates compared to artificial diet (Zhang et al. 2024). In *S. frugiperda*’s OS, there were also three glucose dehydrogenases that were more abundant in the OS from leaf-fed larvae (Zhang et al. 2024). Glucose dehydrogenase catalyzes the oxidation of glucose to gluconolactone by donating electrons to its cofactor FAD. These electrons can be transferred to quinones as electron acceptors and are involved in the generation of reactive oxygen species (Lovallo and Cox-Foster 1999), thus may be related to the reduction of defense metabolites. However, in our study, we found that glucose dehydrogenases were less abundant in the OS of both leaf-fed *S. littoralis* and *S. exigua* larvae (this latter only when fed on pepper leaves) than when fed on artificial diet.

In addition to enzymes, other proteins display differential abundance in both *Spodoptera* species, potentially linked to plant-insect interaction. One of them is polycalin, which was highly abundant in the OS of both species. Its abundance further increased when the diet switched from artificial to plant-based. Polycalin, also known as chlorophyllide A binding protein, belongs to the group of chlorophyll binding proteins (Badgaa et al. 2015). Although lipocalin-type proteins typically lack catalytic properties, the protein complex with chlorophyllide A exhibits broad-spectrum antimicrobial activity (Pandian et al. 2010), potentially enhancing the insect’s resistance when consuming a chlorophyll-rich diet and exposed to light (Angelucci et al. 2008; Campbell et al. 2008). Another protein that exhibited increased abundance in both *Spodoptera* species when fed a lead-based diet, compared to the artificial diet, is the β-1,3-glucan-binding protein. β-1,3-glucanases are enzymes known to be expressed in the hemolymph and fat body of caterpillar larvae, and involved in innate immunity (Fabrick et al. 2004; Li et al. 2022; Ma and Kanost 2000; Takahashi et al. 2014). Specifically, they detect β-1,3-glucan on fungal surfaces via their N-terminal carbohydrate-binding domain (N-βGRP) and trigger serine protease cascades for the activation of prophenoloxidase (Takahashi et al. 2014). The presence of β-1,3-glucan-binding protein in the OS of both *Spodoptera* larvae, which increases when switched from an artificial diet to a plant-based diet is intriguing, and warrants further exploration. Another protein that displayed a consistent pattern of differential abundance in both species and under both plant-based diet treatments compared to the artificial diet is the WD-repeat protein, which decreased in abundance when switching from a plant-based to an artificial diet. WD-repeat proteins play important roles in various cellular functions, including signal transduction, cell cycle control, intracellular transport, development, transcriptional regulation, and immune responses (Jain and Pandey 2018), and the reason for its presence in the OS is intriguing.

Lastly, it is important to mention the group of REPAT proteins, recently identified as effectors of herbivory-induced defense in the noctuids *H. armigera* (referred to as HARP1 and HAS1) (Chen et al. 2019, 2023)d *frugiperda* (referred to as VNPR4) (Zhang et al. 2024). We identified several REPAT proteins in the OS of *S. exigua* and *S. littoralis*, specifically twelve REPATs in *S. exigua* and seven in *S. littoralis*. Among these, the abundance of seven REPATs in *S. exigua* and tree REPATs in *S. littoralis* changes when the larvae switch from a plant-based diet to an artificial one, with some increasing and others decreasing in abundance. REPATs are encoded by a large gene family, with at least 46 isoforms transcribed in *S. exigua* (Navarro-Cerrillo et al. 2013). Their presence in the OS of *Spodoptera* larvae is likely due to their expression in the caterpillars’ gut (Bel et al. 2013; Herrero et al. 2007; Navarro-Cerrillo et al. 2013) though an isoform as also been found in the saliva of *S. frugiperda* (Acevedo et al. 2017). Therefore, the isoforms whose abundance increases when *S. exigua* and *S. littoralis* switch from artificial to plant-based diet may be involved in the modulation of plant defense response like HARP1 in *H. armigera* or VNRP4 in *S. frugiperda*.

In conclusion, our findings highlight the potential role of *Spodoptera* OS proteins in the modulation of plant-larvae relationships. We demonstrated a diet-mediated OS plasticity that may both reflect the adaptation to the digestion of different substrates, such as a differential abundance of several proteases or polycalin, as well as the identification of putative effectors and elicitors conserved across *Spodoptera* species, such as lipases and REPAT proteins, which deserves further investigation to dissect the specific contribution of each isoform involved in these interactions.

## Electronic Supplementary Material

Below is the link to the electronic supplementary material.

Supplementary Material 1

Supplementary Material 2

Supplementary Material 3

Supplementary Material 4

Supplementary Material 5

Supplementary Material 6

## Data Availability

The mass spectrometry proteomics data have been deposited to the ProteomeXchange Consortium with the dataset identifier PXD056472
